# Fuzzy petrology in the origin of carbonatitic/pseudocarbonatitic Ca-rich ultrabasic magma at Polino (central Italy)

**DOI:** 10.1038/s41598-019-45471-x

**Published:** 2019-06-25

**Authors:** Michele Lustrino, Natascia Luciani, Vincenzo Stagno

**Affiliations:** 1 Dipartimento di Scienze della Terra, Sapienza Università di Roma, P.le A. Moro, 5, 00185 Roma, Italy; 2 CNR – Istituto di Geologia Ambientale e Geoingegneria (IGAG) c/o Dipartimento di Scienze della Terra, Sapienza Università di Roma, P.le A. Moro, 5, 00185 Roma, Italy; 3 Faculty of Earth and Life Sciences, Vrije Universiteit Amsterdam, de Boelelaan 1085, 1081 HV Amsterdam, The Netherlands

**Keywords:** Geochemistry, Petrology

## Abstract

The small upper Pleistocene diatreme of Polino (central Italy) is known in literature as one of the few monticellite alvikites (volcanic Ca-carbonatite) worldwide. This outcrop belongs to the Umbria-Latium Ultra-alkaline District (ULUD), an area characterized by scattered and small-volume strongly SiO_2_-undersaturated ultrabasic igneous rocks located in the axial sector of the Apennine Mts. in central Italy. Petrographic and mineralogical evidences indicate that Polino olivine and phlogopite are liquidus phases rather than mantle xenocrysts as instead reported in literature. The presence of monticellite as rim of olivine phenocrysts and as groundmass phase indicates its late appearance in magma chambers at shallow depths, as demonstrated by experimental studies too. The absence of plagioclase and clinopyroxene along with the extremely MgO-rich composition of olivine (Fo_92–94_) and phlogopite (average Mg# ~93) suggest for Polino magmas an origin from a carbonated H_2_O-bearing mantle source at depths at least of 90–100 km, in the magnesite stability field. In contrast with what reported in literature, the ultimate strongly ultrabasic Ca-rich whole-rock composition (~15–25 wt% SiO_2_, ~31–40 wt% CaO) and the abundant modal groundmass calcite are not pristine features of Polino magma. We propose that the observed mineral assemblage and whole-rock compositions result mostly from the assimilation of limestones by an ultrabasic melt at a depth of ~5 km. A reaction involving liquidus olivine + limestone producing monticellite + CO_2_ vapour + calcite is at the base of the origin of the Polino pseudocarbonatitic igneous rocks.

## Introduction

**Figure 1:**
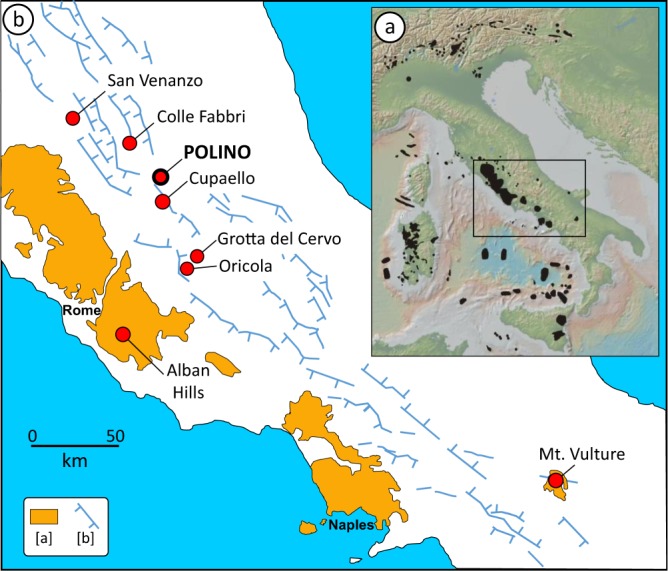
(**a**) Occurrence of the main subaerial and submerged Cenozoic igneous districts of circum-Tyrrhenian and Alpine realms. (**b**) Simplified geological map showing the main outcrops of carbonatites of Italy reported in literature. Red circles indicate ULUD outcrops, carbonatites and strongly SiO_2_-undersaturated ultrapotassic volcanic rocks some of which as root-less pyroclastic facies. Alban Hills and Mt. Vulture are volcanoes where carbonatites or magmatic calcite have been reported. a = Plio-Quaternary potassic to ultrapotassic volcanic rocks; b = normal faults.

The axial portion of the Apennine fold-and-thrust belt in central Italy is characterized by the sporadic presence of upper Pleistocene low-volume alkali- and lime-rich igneous rocks belonging to the kamafugite-melilitite-carbonatite clan^1–4^, erupted along fault systems bordering Plio-Pleistocene grabens. Two of these outcrops near San Venanzo and Cupaello villages are type localities of strongly SiO_2_-undersaturated kalsilite-bearing rocks named venanzite (a melanocratic variety of leucite olivine kalsilite melilitite) and coppaelite (a phlogopite kalsilite pyroxene melilitite without olivine)^5^. A ~10–15 m wide diatreme of Ca-rich rocks crops out in the Polino village in between San Venanzo and Cupaello (Fig. 1). These rocks, dated with ^40^Ar/^39^Ar ~246 ka^6^, so far have been classified as monticellite alvikites, i.e., volcanic Ca-carbonatites^7–10^.

The identification of Ca-carbonatitic volcanic activity has been considered a key feature to unravel the geodynamic setting of the Plio-Quaternary volcanism of central Italy. In particular, the presence of carbonatitic magmas has been proposed to deny the existence of active or fossil subduction tectonic settings along peninsular Italy, proposing the existence of a deep seated variably asymmetric (eastward-pushing) mantle plume or hot spot^1,11–13^. From a general point of view, “*a strong evidence for the link between the carbonatite genesis and the locations of deep-mantle plumes*” has been recently proposed^14^.

The mantle source of the calcite-, melilite- and kalsilite-bearing rocks is assumed to be the same of the potassic and ultrapotassic volcanic rocks of central-southern Italy (Latium-Campania administrative regions^11^). On this ground, any subduction-related process has been considered highly improbable for the entire Plio-Quaternary volcanism of peninsular Italy too (e.g., refs^13,15^).

On the other hand, the majority of researchers considers the potassic and ultrapotassic volcanic rocks of peninsular Italy (including the rare kamafugitic-melilititic sub-groups) as occurring in a subduction-related setting, interpreting the lime-rich compositions like hybrid melts or paralavas (e.g., refs^4,16–21^). The Pleistocene carbonatitic lava flow (alvikite) found at Mt. Vulture are interpreted as generated from liquid unmixing at crustal levels starting from nephelinitic to melilititic parental magmas, these latter generated above a slab window in a metasomatized mantle wedge^22^.

The aim of this work was to investigate the origin of Polino volcanic rocks (Terni, Italy), so far classified as Ca-carbonatite because of their abundance of calcite and overall Ca-rich and Si-poor whole-rock composition. The coexistence of Mg-rich olivine, Mg-rich phlogopite, monticellite, globular and sparry calcite and the absence of feldspars and pyroxenes make Polino rocks unique worldwide, distinct from the other rare cases of monticellite carbonatites reported in literature.

On the basis of petrographic, mineral chemical and whole-rock geochemical evidences, as well as petrologic considerations, we propose an alternative petrogenetic process. Our main conclusions can be summarized as: (1) olivine and phlogopite macrocrysts are liquidus phases rather than mantle debris; (2) the absence of plagioclase and pyroxene along with the high Mg# of olivine and phlogopite indicate strongly SiO_2_-undersaturated magma compositions (CIPW larnite normative) formed by melting of a magnesite-bearing peridotitic mantle; (3) the actual mineral assemblage of Polino rocks consisting of olivine, phlogopite, monticellite and calcite can be explained in light of shallow-depth digestion of country rocks represented by a ~5 km-thick limestone succession; (4) the Polino igneous rocks are better classified as pseudocarbonatites or, more properly, endoskarns; (5) carbonatites, commonly considered in literature to be indicative of within-plate or continental rifting settings, connected to the presence of mantle plumes^13,14,23,24^, cannot be used to infer geodynamic environments.

## Carbonatites

Carbonatites are igneous rocks composed of >50% modal primary carbonate and with <20 wt% SiO_2_ (ref.^5^). Carbon must be juvenile – magma-derived – and not from wall rocks or external hydrothermal fluids. Calcite-carbonatite (also defined as calcio-carbonatite) magmas are by far the most abundant lithologies of this extremely rare rock group. Carbonatites can be either alkali-rich or alkali-poor, and are commonly associated with silicate rocks characterized by alkaline ultrabasic compositions such as nephelinite/nephelinolite, melilitite/melilitolite, essexite, aillikite, melteigite and kimberlite, but can also be genetically related to evolved and SiO_2_-richer lithologies such as trachyte/syenite, phonolite/nepheline syenite^25^. Albeit rare^26^, carbonatitic inclusions have been reported in mantle xenoliths found in mafic sodic magmas (e.g., refs^27,28^). On the other hand, mantle xenoliths have been never^29^ or rarely^30^ recorded in carbonatitic magmas because of their preferential settling in low viscosity and low density magmas.

The origin, the evolution and the plate tectonics significance of carbonatites have been debated for decades with several models proposed over the last 50 years. Mitchell (ref.^31^) described three main types of carbonatites: (*1*) Primary Carbonatites – genetically related to mantle-derived magmas such as nephelinite, melilitite and kimberlite. These magmas form by partial melting of a carbonated peridotite or eclogite (e.g., ref.^32^), likely followed by fractional crystallization (e.g., ref.^33^), and liquid immiscibility from alkali-rich to alkali-poor silicate magmas (e.g., refs^29,34–36^); (*2*) Carbothermal Carbonatites^37^ – related to low-temperature fluids derived from fractionated magmas enriched in CO_2_, H_2_O and fluorine; (*3*) Pseudocarbonatites – formed by anatectic melting of crustal rocks, also named calc-silicate skarns or anatectic calc-silicate veins. Barker (ref.^29^) also considers the problem of pseudocarbonatites, defining them carbonate-rich rocks with hydrothermal, metamorphic or sedimentary origin, to be distinguished by true igneous carbonatites. Lentz (ref.^38^) considers pseudocarbonatites in terms of skarn reaction equilibria too.

Gudfinnsson and Presnall (ref.^39^) showed experimentally that the composition of melts generated by melting of a carbonated peridotite is strictly related to the temperature and depth of the source rock. With increasing temperature, between 2 and 8 GPa, different melts can be produced, with compositions ranging from carbonatitic (<10 wt% SiO_2_; CaO >25 wt%) to melilititic (~20–30 wt% SiO_2_; CaO ~20–25 wt%), kimberlitic (~30–40 wt% SiO_2_; CaO <20 wt%) and eventually basaltic (~>40 wt% SiO_2_; CaO <10 wt%). Below the solidus, the stable carbonate is dolomite at P ~2–3 GPa, and magnesite at P >3 GPa. Carbonatitic melts generated from a carbonated peridotite contain dolomitic to magnesitic phases rather than calcite/aragonite as instead observed in case of melting of Ca-rich eclogite assemblages^40^.

As concerns the circum-Mediterranean area, the presence of carbonatitic rocks (or carbonatitic components) have been reported in SE Libya (Uwaynat), NW Morocco (Taouirt, Tamazert, Saghro), central Spain (Calatrava), central France (Chabrieres, Clermont Ferrand), Germany (Kaiserstuhl, Eifel), Bohemian Massif (Czech Republic, Flurbuhl) eastern and central Anatolia (Malatya, Sivas, Kizilcaoren) and in central and southern Italy (Polino, Alban Hills, Mt. Vulture; ref.^41^ and references therein).

One of the major problems in identifying true carbonatitic rocks is to distinguish magmatic textures from later replacement and secondary textures (e.g., ref.^42^). Indeed, subsolidus plastic flow, deuteric alteration and solution-precipitation recrystallization of magmatic calcite can lead to a difficult distinction from hydrothermal and metasedimentary carbonate rocks^29^. Also the presence of minerals typical of carbonatite rocks cannot be considered a proof for their mantle origin, being these phases also found in calc-skarns^38,43^. We report petrographic and mineralogical evidences according which the carbonate-rich fraction of Polino rocks cannot be considered a feature of primary– i.e., in equilibrium with mantle rocks – conditions.

## Carbonatites in Italy

Rocks interpreted as carbonatites crop out in central and southern Italy (Fig. 1) as carbonatites and carbonatite-related lithologies (Polino, Cupaello, Colle Fabbri, Oricola and San Venanzo) of the *Umbria-Latium Ultra-alkaline District* (ULUD; ~0.64–0.26 Ma) also known as *Intra-Apennine Province* (IAP), *Intra-Apennine Volcanism* (IAV) or *Intramontane Ultra-alkaline Province* (IUP^1,4,6,11,44–47^). These rocks are sometimes associated with strongly SiO_2_-undersaturated melts such as melilitites and kamafugites, as well as with minor evolved compositions (trachyphonolites); only in the Polino case no associated silicatic magma is present being the monticellite alvikite the only reported igneous rock. The typical (but not ubiquitous) mineral paragenesis of ULUD rocks is represented by variable amounts of foids (mostly leucite and nepheline-kalsilite solid solution), melilite, phlogopite, olivine and clinopyroxene.

Southern Italy carbonatites are reported in Mt. Vulture volcanic complex as a small alvikitic lava flow^22^, as plutonic xenoliths (sövite^48^) and as carbonate inclusions in mantle xenoliths^49^. High δ^18^O clinopyroxenes (up to +8.3‰), the correlation between δ^18^O and Si-Al^IV^ content in clinopyroxene, Mg#-rich clinopyroxene (up to 95) and phlogopite (up to 93), Fo-rich olivine (up to 98), abundant skarn xenoliths, high-energy explosive eruptions (e.g., Villa Senni) and groundmass calcite in Alban Hills lavas and granular ejecta are interpreted as a result of assimilation of limestone wall rocks by basic primary melts (e.g., refs^43,50–55^). This is modelled as open system skarnification of a basic silicatic melt which digested limestone walls of a shallow magma chamber, evolved via fractional crystallization towards the silicate-carbonatite solvus, generating carbonatitic melts with clear secondary origin^54^.

Carbonatites from central Italy are associated to small monogenetic centres (diatreme structures) pyroclastic rocks and minor lavas with ultrapotassic composition located close to the Apennine Chain axis (Fig. 1). Overall, the trace element content and Sr-Nd-Pb isotopic ratios of Italian carbonatites match those of the Roman Province rocks^47^, but their origin remains highly debated. Stoppa *et al*. (ref.^56^) interpret the carbonate-rich lithologies associated with the ultrapotassic rocks as true Ca-carbonatitic melts, while Peccerillo (ref.^57^) relates them to interaction between silicate melt and sedimentary limestone country rocks.

According to several authors, the volcanic rocks of Polino, classified as monticellite calcio-carbonatites^7–10,45,58^, are characterized by abundant mantle xenoliths and xenocrysts (forsterite and phlogopite). Here, we demonstrate that the petrographic features, the mineralogy and the chemical composition of Polino ultrabasic rocks are more compatible with shallow depth assimilation of sedimentary carbonate wall rocks by SiO_2_-poor Mg-rich carbonated melts.

## Polino

**Figure 2:**
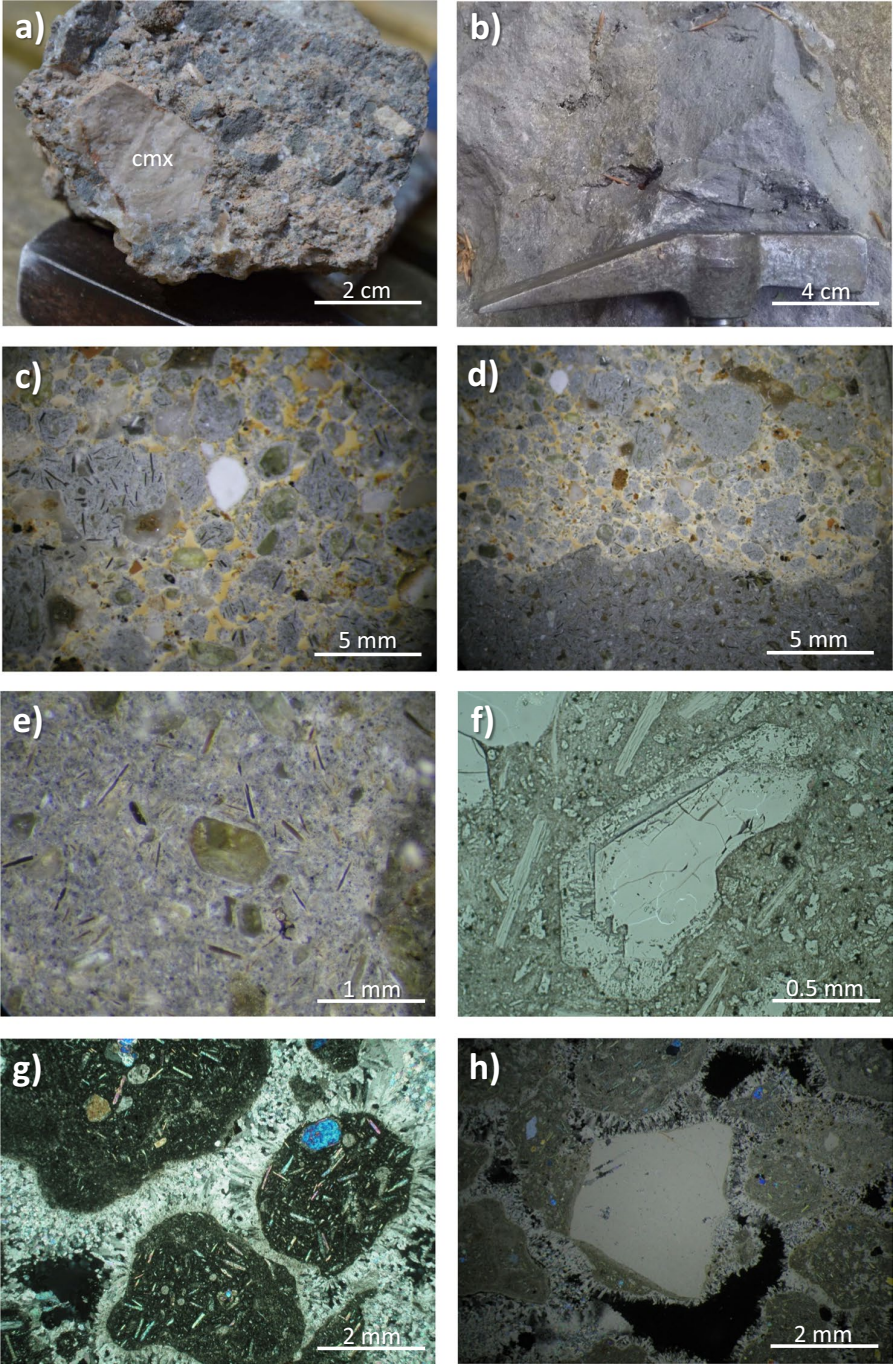
Polino volcanic rocks. (**a**) Pyroclastic facies with a large Calcare Massiccio limestone xenolith (cmx), smaller melt droplets (greyish) and smaller limestone xenoliths agglutinated in an havana colour calcitic cement. (**b**) Massive facies. (**c**) Close-up of polished pyroclastic facies with silicatic melt droplets (greyish) agglutinated by sparry calcitic cement (yellowish to brownish) and Calcare Massiccio limestone xenoliths (white) with olivine (greenish) and phlogopite (acicular habit) phenocrysts. (**d**) Close up of polished contact between pyroclastic (top) and massive (bottom) facies. Only the massive facies samples have been selected for whole-rock analyses. (**e**) Close up of polished massive facies with euhedral olivine and phlogopite phenocrysts. (**f**) Plane-polarized view of euhedral olivine phenoclast rimmed by monticellite rim, set, together with phlogopite phenocrysts and micro-phenocrysts, in a fine-grained monticellite- and calcite-rich groundmass. (**g**) Crossed polar view showing silicatic globules – characterized by high-birefringent olivine and phlogopite phenocrysts and havana calcite globules – cemented by sparry radiating calcite. (**h**) Crossed polar view showing a large Calcare Massiccio limestone xenolith in a scoriaceous (black = voids) pyroclastic facies with silicatic globules cemented by sparry radiating calcite.

Despite the limited outcrop (<100 m^2^), the Polino volcanic rocks occur in two facies. The first (pyroclastic facies) is characterized by the presence of mm- to cm-sized droplets of silicate melt agglutinated by sparry calcite cement, while the second show a more massive aspect (Fig. 2). Both facies are characterized by abundant euhedral to subhedral forsteritic olivine (Fo_92–94_) followed by euhedral phlogopite (Mg# 90–94) and monticellite, coexisting with accessory phases as Ca-Ti/Ca-Si perovskite, schorlomitic garnet and Fe-Ti oxides, all associated with variable amounts of sparry, sedimentary or ameboid calcite (e.g., ref.^59^; Fig. 2). All the minerals are SiO_2_-poor to SiO_2_-free phases, i.e., to complete occupancy of tetrahedral sites in the schorlomitic garnet, all the Al and half of Fe must be assigned to tetrahedral coordination^59^. These rocks are characterized by the absence of feldspars and pyroxenes, with the exception of very rare (<0.1% vol) occurrences of sanidine and diopside xenocrysts from older phonolitic pyroclastic cover pierced by the diatreme^8^. The Polino diatreme crosses the Calcare Massiccio Formation, a Lower Jurassic peritidal carbonate succession cropping out in the Apennines originally deposited along the passive margin of the Adria micro-plate (e.g., ref.^60^). During two campaigns in 2016 and 2017, twelve samples were collected and analysed for petrographic investigation using the polarizing microscope, scanning electron microscopy (SEM) and electron microprobe (EMP), while three of them have been analyzed for major oxides (ICP-AES) and trace elements (ICP-MS) at the Activation Laboratories (Ontario, Canada). Only massive facies samples have been chosen for analyses, excluding pyroclastic facies rocks rich in sparry calcite cementing silicate globules. About 0.2 g sample have been thermally decomposed in a resistance furnace in a pure nitrogen environment at 1000 °C, directly releasing CO_2_. H_2_O is removed in a moisture trap prior to the detection of CO_2_ in the infra-red cell. Full details in http://www.actlabs.com. The EMP details are reported in Lustrino *et al*. (ref.^61^).

**Figure 3:**
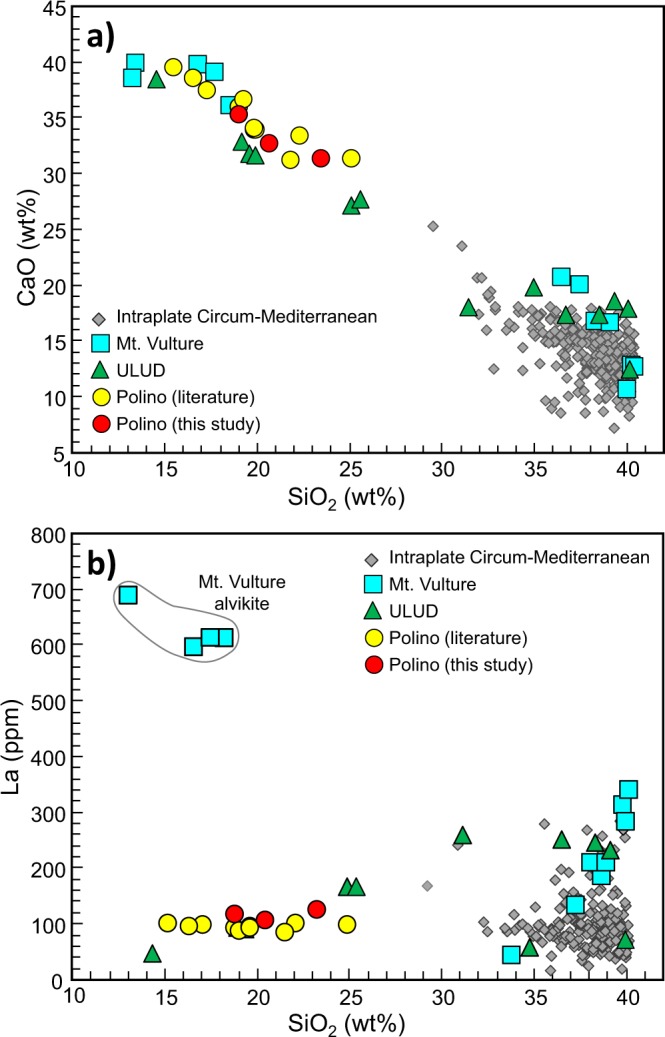
CaO vs. SiO_2_ (**a**) and La vs. SiO_2_ (**b**) whole-rock composition of Polino volcanic rocks. The composition of other ULUD (Umbria-Latium Ultra-alkaline District) rocks, as well as Mt. Vulture and circum-Mediterranean igneous rocks with SiO_2_ <40 wt% are reported for comparison (ref.^21^ and references therein).

**Figure 4:**
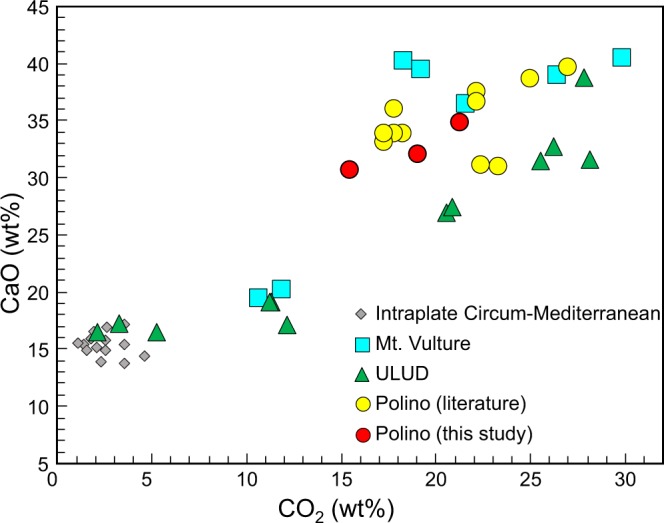
CaO vs. CO_2_ whole-rock composition of Polino volcanic rocks. The composition of other ULUD (Umbria-Latium Ultra-alkaline District) rocks, as well as Mt. Vulture and circum-Mediterranean igneous rocks with SiO_2_ <40 wt% are reported for comparison (ref.^21^ and references therein).

Some of the major oxides of the three Polino volcanic rocks analyzed in this study (full dataset in Table A1) are reported in Figs 3 and 4, together with the Polino literature data, the composition of the ULUD, Mt. Vulture rocks and other intraplate circum-Mediterranean ultrabasic rocks with SiO_2_ content <40 wt%. The LOI of the Polino rocks is very high (19.8–25.2 wt%), as expected for calcite-rich compositions.

The rocks analyzed in this study show low SiO_2_ contents (~18.8–23.8 wt%), within the composition reported in literature of ~15.1–24.8 wt% (refs^7,8,45^). The range in silica content is surprisingly wide considering the outcrop extension (<100 m^2^) and is here explained as due to the difficulty to completely separate the sparry cement from the silicatic portion in the pyroclastic facies rocks. The CaO content ranges from 31.6 to 39.7 wt% and shows negative correlation with SiO_2_ (R^2^ = 0.89). With the exception of two literature analyses, CaO is correlated with CO_2_ (14.5–26.0 wt%; R^2^ = 0.81; Fig. 4). These correlations highlight two components, one relatively SiO_2_-rich but CaO- and CO_2_-poor, and the other SiO_2_-poor (to SiO_2_-free) and Ca-CO_2_-rich. The second term has been considered the most representative Italian carbonatite end-member by Martin *et al*. (ref.^10^) and is reported in several worldwide carbonatite lists (e.g., refs^14,29,30,62–64^). Polino rocks are alkali-poor, with Na_2_O <0.15 wt% and K_2_O <0.92 wt%, a feature in contrast with the coeval and nearby potassic to ultrapotassic composition of the Italian Pleistocene volcanic rocks and the other ULUD products^4,20,21,42^.

Compared with the average Ca-carbonatite compositions^65,66^, the average composition of Polino rocks shows much lower Sr (1690 ppm vs. 7272 ppm), Nb (15 ppm vs. 343 ppm), Ta (0.7 ppm vs. 9.1 ppm) and LREE (e.g., Ce = 217 ppm vs. 1687 ppm), coupled with much higher Rb (63 ppm vs. 14 ppm), Cr (526 ppm vs. 13 ppm) and Ni (320 ppm vs. 18 ppm). A comparison with natural monticellite carbonatites shows the peculiarity of Polino rocks. Indeed, Polino rocks, compared with Fort Portal (Uganda) monticellite Ca-carbonatite lavas and tuffs, have lower TiO_2_ (<0.6 wt% vs. >1.6 wt%), Fe_2_O_3tot_ (<5.3 wt% vs. >9.0 wt%), Na_2_O (<0.19 wt% vs. >0.37 wt%), P_2_O_5_ (<0.6 wt% vs. >1.6 wt%), V (<55 ppm vs. >207 ppm), Sr (<1897 ppm vs. >3324 ppm), La (<122 ppm vs. >250 ppm), Nb (<21 ppm vs. >241 ppm) and Ta (<0.8 ppm vs. >10.6 ppm), as well as higher Cr (>368 ppm vs. <142 ppm) and Ni (>231 ppm vs. <73 ppm). Moreover, Fort Portal carbonatitic lavas and tuffs are characterized by the association of Mg-rich calcite (MgO up to 13 wt%) and calcite, while Polino rocks host Mg-poor calcites (MgO <0.6 wt%) only. Fort Portal carbonatites are also associated to abundant silicatic magma (mostly melilitites), while in Polino no associated silicate rocks are present (being the silicatic component of Polino interpreted in literature as related to the contamination of a pure Ca-carbonatitic melt with matrix peridotite *en route* to the surface).

## Whole Rock and Mineral Chemical Constraints for the Origin of Polino Rocks

The relationship between olivine and monticellite is the key aspect to unravel the origin of the Polino magma. Polino monticellite has been interpreted as the result of the interaction between a Ca-carbonatitic mantle melt with xenocrystic mantle forsterite. According to several authors (refs^7–9,58^), the Ca-carbonatitic melt should be generated at mantle depths by immiscibility from a carbonatite-melilitite liquid. Then, it partially reacted with portions of mantle wall rocks during its ascent to the surface, with the chemical interaction between liquid carbonatite and mantle olivine being expressed in the form^9^:

1 $${{\rm{CaCO}}}_{3({\rm{melt}})}+{{\rm{Mg}}}_{2}{{\rm{SiO}}}_{4({\rm{xenocryst}}{\rm{mantle}}{\rm{olivine}})}={{\rm{CaMgSiO}}}_{4({\rm{monticellite}})}+{{\rm{MgO}}}_{({\rm{melt}})}+{{\rm{CO}}}_{2({\rm{vapour}})}$$

This reaction would result in a dilution of the original CaO content of the carbonatitic magma coupled with an increase of its SiO_2_ and MgO content. According to Bell and Kjarsgaard (ref.^67^; p. 86) the presence of mantle xenoliths at San Venanzo and Polino “*is a testament to the rapid ascent of the kamafugitic and carbonatitic magmas*”, rendering “*highly problematic*” an open system interaction between a silicatic (kamafugitic) magma and local limestone. On the other hand, Barker (ref.^29^; p. 46) underlines that ultramafic mantle xenoliths have never been reported in carbonatite lavas or intrusions, due to “*the low viscosity and low density of carbonatitic liquid or to the separation of an immiscible liquid from mantle-derived silicate magma within a crustal reservoir*. *In both cases*, *ultramafic xenoliths would settle out*”.

Fort Portal carbonatite contains a variable cargo of silicatic rocks as gneissic, amphibolitic, and gabbroic xenoliths. In order to calculate the relatively pristine composition of the carbonatitic magma, Eby *et al*. (ref.^68^), following the approach of Barker and Nixon (ref.^69^), subtracted the silicatic component by regressing major and trace elements vs. alumina to Al_2_O_3_ = 0. In many cases (SiO_2_, TiO_2_, MnO, P_2_O_5_, Cr, Ni, Zn, REE, Y, Nb, Zr, Ta, Th) the R^2^ value obtained with these regressions is very high (>0.9), indicating the petrological sound of such a procedure. If olivine and phlogopite in Polino rocks are mantle xenocrysts, we should obtain similar results. However, a regression of both major and trace elements vs. Al_2_O_3_ do not show any appreciable correlation, with major elements showing R^2^ values <0.39 and trace elements showing R^2^ values <0.33.

**Figure 5:**
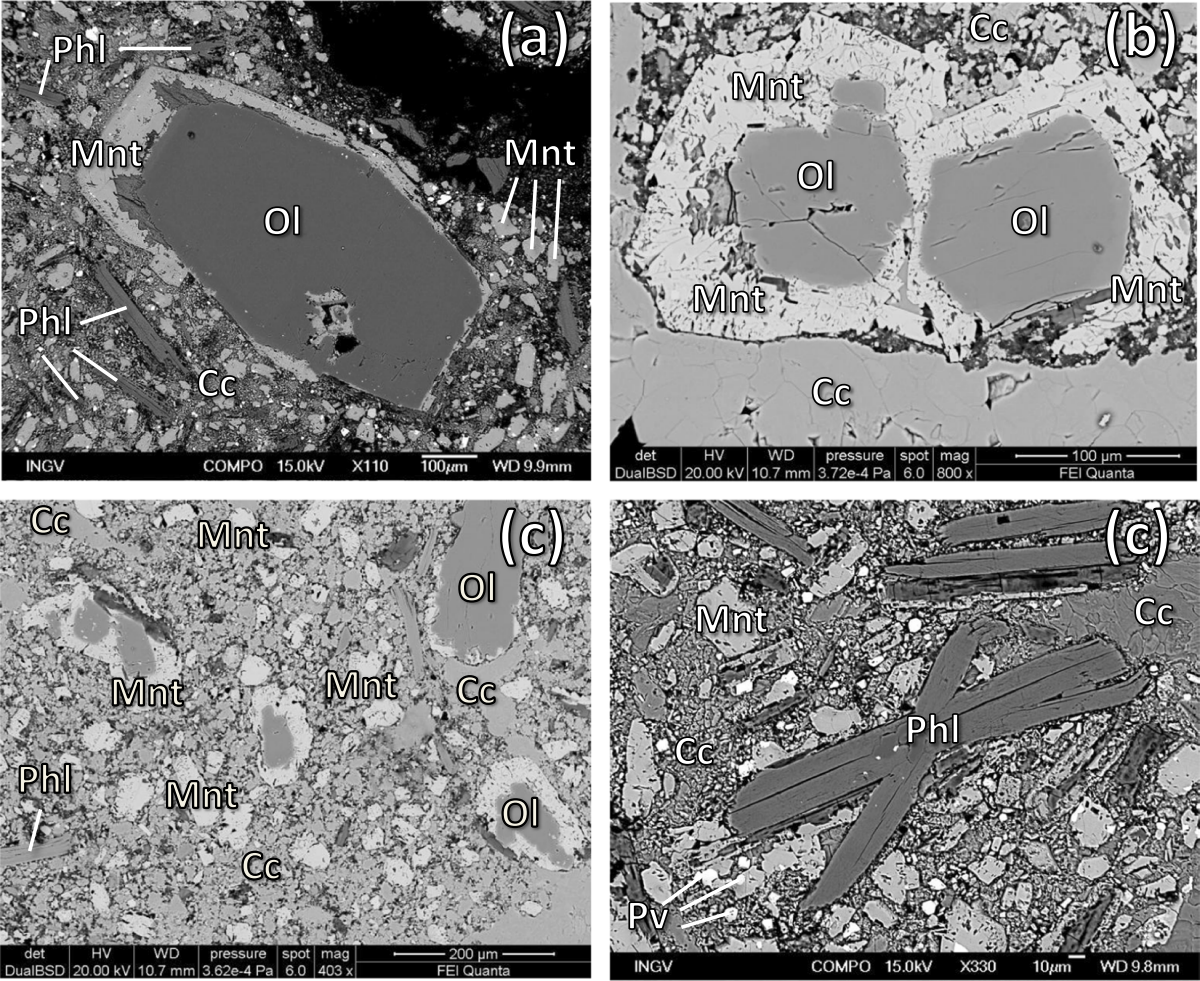
(**a**) SEM images of Polino euhedral olivine (Ol) rimmed by monticellite (Mnt), groundmass monticellite, euhedral phlogopite (Phl) and ameboid calcite (Cc). (**b**) SEM image of Polino euhedral olivine rimmed by monticellite close to a border of sparry calcite. (**c**) SEM image of Polino rock petrography. (**d**) SEM image of Polino euhedral phlogopite laths in a groundmass made up of monticellite, calcite and perovskite (Pv).

A microstructural and compositional study of the main mineral phases found in Polino rocks allows us to exclude a xenocrystic origin for forsterite and phlogopite. These two minerals, indeed, are characterized by euhedral to subhedral shape, with phlogopite mostly represented by tiny elongated euhedral laths (Figs 2 and 5; see also pictures in Table A2). Interestingly, the age of Polino rocks (~246 ka) has been estimated via ^40^Ar/^39^Ar on phlogopite separates, indicating that this is a phase crystallized from a melt rather than a mantle xenocryst.

The euhedral shape strongly differs from olivine crystals found in mantle xenoliths (but also in Alpine-type ophiolitic massifs or dredged peridotites), always characterized by anhedral habit. The interpretation of Polino olivines as mantle xenocrysts seems, therefore, highly improbable (e.g., ref.^70^). In addition, the absence of any deformation texture in the olivine crystals in Polino olivines is a further proof at odd with a mantle xenocrystic origin hypothesis, supporting the derivation as a liquidus phase. The lack of peridotite mantle xenoliths or mantle xenocrysts would be sufficient to confute the reaction (1).

**Figure 6:**
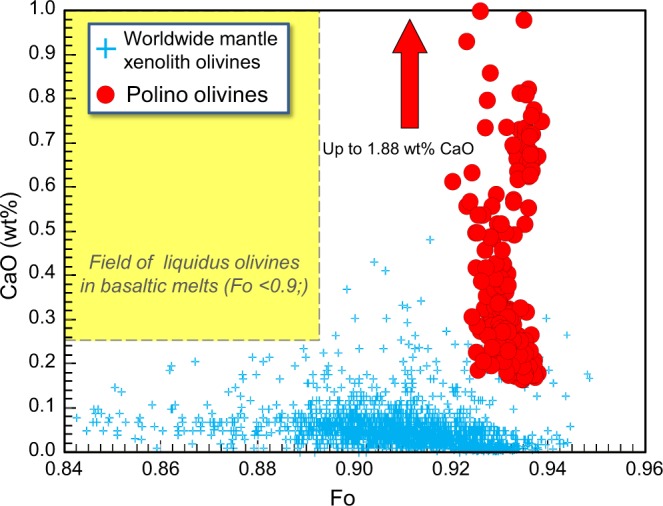
CaO vs. Fo diagram for Polino olivines. The analyses of worldwide mantle xenolith olivines taken from PEDTB database are reported for comparison.

**Figure 7:**
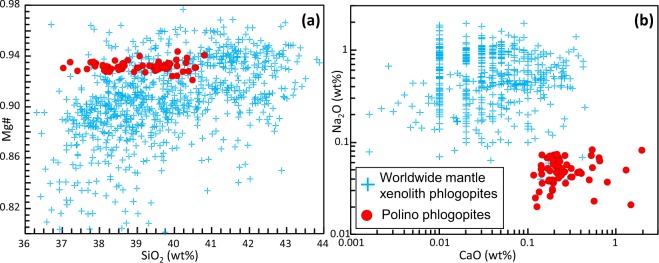
Polino phlogopite EMP data. Worldwide phlogopites in mantle xenoliths from GEOROC database, selecting rock types falling in dunite, harzburgite, lherzolite and peridotite definitions and with the following additional filters: TiO_2_ <10 wt%, Al_2_O_3_ >9 wt%, Cr_2_O_3_ <3 wt%; MgO >10 wt% and <30 wt%, Na_2_O <2 wt%, Mg# >0.80). The complete list can be requested to the first author.

Our new interpretation of Polino olivines as liquidus phase is also supported by EMP data. The forsterite content of olivine (expressed as Fo-Fa solid solution) is particularly high, mostly clustering in the 91.8–94.0 range (463 analyses; average = 93.2, st. dev. ±0.34; Table A2), much higher than the Mg# of monticellite (75.8–85.1; 83 analyses; average 79.2, st. dev. ±2.3; Table A2). This high forsterite content has been considered the strongest proof for a mantle xenocrystic origin (e.g., refs^7–9,58^). In the CaO vs. Fo diagram (Fig. 6), the Polino olivines are compared with worldwide mantle xenolith olivines from the PETDB database (https://www.earthchem.org/petdb). Polino olivines are distinct, being characterized by much higher CaO content (0.16–1.9 wt%) than mantle olivine (<0.12 wt% CaO for >95% of the data). The major element content of Polino olivines also reflects their liquidus origin, albeit the anomalously high Fo content requires specific interpretation (see below). Similarly, phlogopite is characterized by Mg-rich compositions with Mg# ratios (Mg# = 100 * [Mg/(Mg + Fe^2+^)]) ranging from 90.1 to 94.5 (59 analyses; average 93.19, st. dev. ±0.39; Fig. 7a and Table A3), but plot far from the field of typical mantle phlogopites when minor elements are taken into account (e.g., Na_2_O vs. CaO; Fig. 7b).

Polino olivines are characterized by relatively high Ni (ranging from ~550 to ~5700 ppm; average = ~3300 ppm, st. dev. ±~1180 ppm; Table A2). According to Ammannati *et al*. (ref.^71^), the Italian plagio-leucititic rocks have olivine poor in Ni (~1100–1960 ppm; average ~1330 ppm) because generated from a peridotitic mantle metasomatized by CaCO_3_-rich agents. On the other hand, SiO_2_-richer lamproitic rocks are characterized by much higher Ni (~1960–4950 ppm; average ~3140 ppm). The reason of Ni enrichment in olivine from lamproitic melts would be the derivation of these liquids from olivine-poor to olivine-free mantle source. The depletion in olivine would result after the reaction of mantle forsterite with silica-rich agents released from recycled terrigenous sediments, forming enstatite-rich metasomes^72,73^. On the other hand, the low-Ni (and high-Ca; ~2400–4000 ppm) content of olivines in the plagio-leucitites would reflect “*the effect of the reaction between melts of carbonate-rich sediments with peridotite*, *stabilizing newly formed olivine and clinopyroxene at the expense of orthopyroxene*” (ref.^71^; p. 72). Polino olivines do not have the low Ni content of the melts generated from a carbonate-metasomatized sources, but share with them the relatively Ca-rich (~430–6220 ppm; average ~2720 ppm) content. This questions the validity of the general rule proposed by Ammannati *et al*. (ref.^71^).

The extremely MgO-rich composition of olivine up to Fo_94_ and phlogopite up to Mg# 94, can be related either to: (1) a strongly depleted (i.e., Fe-poor) mantle source; (2) Fe-Mg distribution coefficient between liquidus olivine and coexisting primitive melt [Kd(Fe-Mg)_ol/melt_] much lower than what measured in melts produced from CO_2_-free peridotitic source with Kd(Fe-Mg)_ol/melt_ of 0.30 ( ± 0.03; ref.^74^); (3) redox conditions resulting after CaCO_3_ digestion, under which Fe^2+^ is oxidised to Fe^3+^, hence limiting its incorporation into the olivine lattice (e.g., refs^53,72^); (4) digestion of basement dolomitic rocks^75^; (5) anomalously Mg-rich (e.g., dolomite/magnesite-bearing carbonated mantle) source.

**Figure 8:**
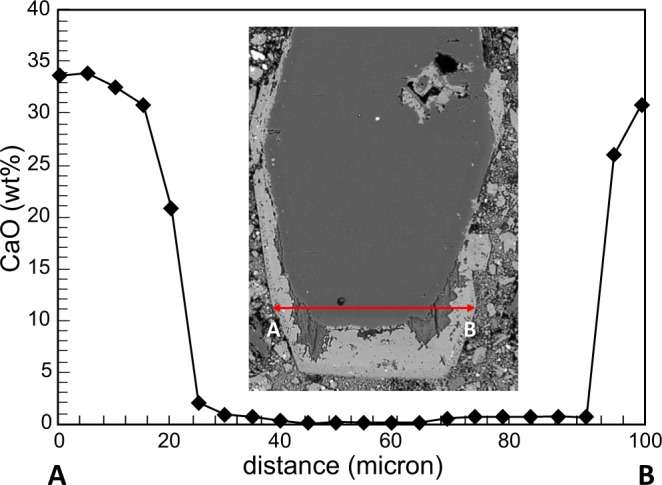
CaO transect of a compositionally homogeneous Polino olivine phenocryst rimmed by a thin monticellite rim seen in BSE and analyzed with EMP.

Forsterite is never observed in the groundmass, but as phenocryst only, characterized by a variably thick monticellite rim. The chemical composition of Polino olivine is, also in the largest crystals, homogeneous (Table A2), suggesting that the melt from which the euhedral olivine crystallized was chemically homogeneous and did not change over time. An abrupt compositional variation occurs in the outer rims only, when CaO content sharply increases from values usually <0.8 wt% to >31 wt%, typical of monticellite, coupled with MgO decrease from >50 wt% to <20 wt%, only few microns across (Fig. 8; Table A2). The boundary between olivine and monticellite rim appears always abrupt and corroded, with signs of reaction and irregular contacts. This can be interpreted as a chemical shock in the form of a change of composition of the crystallizing melt.

We interpret this feature as the indicator of the external input of CaO-rich lithologies in the silicatic magma when it started to pond at shallow crustal depths within sedimentary carbonate magma chamber at a pressure of ~200 MPa. During shallow depth ponding, the hydrous (because phlogopite-bearing) silicatic magma transferred latent heat of crystallization to the country rock, allowing partial dissolution of sedimentary calcite, strongly enriching the melt with CaO and CO_2_ formed by calcite breakdown. Worth noting, the upwelling magma at Polino had to pierce a Jurassic dolostone and limestone succession ~5 km thick^76^, and it is hard to believe that such a small volume of magma can have escaped significant interaction with wall rocks.

A peculiarity of Polino rocks is the common presence of phlogopite despite the low whole-rock K_2_O content (average 0.63 wt% ±0.21 wt%; Table A1). The high SiO_2_ content of sanidine (~65 wt%) prevents its formation in strongly SiO_2_-undersaturated melts such as those of Polino. Alternatively, anhydrous strongly ultrabasic K_2_O-rich magma could stabilize kalsilite and/or leucite as the most stable K-bearing minerals. The absence of these two minerals is related to the overall low amount of K_2_O in the magma, at odds with the high K_2_O content of leucite (~22 wt%) and kalsilite (~30 wt%). The presence of dissolved H_2_O and high MgO contents in the magma allowed the formation of phlogopite (~8.8–10.5 wt% K_2_O) as the most stable K-bearing phase. The presence of this mica is, therefore, not to be considered as an anomalous feature, due to the specific conditions of the Mg-rich parental magma (before the interaction with limestones at shallow depths) and the availability of water in the magma.

## Carbonate-Silicate Magma Interaction Styles

Based on textural and chemical evidences, we propose a two-stage process to explain the origin of olivine and monticellite. The first step includes the growth of compositionally homogeneous euhedral forsterite crystals as liquidus phases in equilibrium with an H_2_O- and CO_2_-bearing ultrabasic melt, followed by crystallization of phlogopite at water-saturated conditions. The second step is associated to an abrupt change of composition of the melt manifested by the appearance of monticellite at the expenses of forsterite around olivine phenocrysts and as groundmass phase not associated to any contemporary olivine crystallization. Once monticellite appeared, olivine stopped to grow. Groundmass monticellite is closely associated with precipitation of microcrystalline calcite after assimilation of limestone wall rocks.

An important contribution over the last ten years has come from experimental studies on the melt composition after assimilation of carbonates. All the previous studies have used “basaltic” compositions (i.e., plagioclase-bearing melts) as starting materials to reproduce assimilation paths as function of temperature, pressure and *f*O_2_. These experiments agree in the occurrence of olivine with relatively high Mg content (Mg# ~95) followed by the crystallization of clinopyroxene as result of the magma interaction with limestones according to the reactions:

2 $${{\rm{CaCO}}}_{3({\rm{limestone}})}+2{{\rm{SiO}}}_{2({\rm{melt}})}+{{\rm{MgO}}}_{({\rm{melt}})}={{\rm{CaMgSi}}}_{2}{{\rm{O}}}_{6({\rm{cpx}})}+{{\rm{CO}}}_{2({\rm{vapour}})}$$

3 $$2{{\rm{CaCO}}}_{3({\rm{limestone}})}+{{\rm{Mg}}}_{2}{{\rm{SiO}}}_{4({\rm{olivine}})}+3{{\rm{SiO}}}_{2({\rm{melt}})}=2{{\rm{CaMgSi}}}_{2}{{\rm{O}}}_{6({\rm{cpx}})}+2{{\rm{CO}}}_{2({\rm{vapour}})}$$

4 $$\begin{array}{c}{{\rm{CaCO}}}_{3({\rm{limestone}})}+{{\rm{SiO}}}_{2({\rm{melt}})}+{{\rm{MgO}}}_{({\rm{melt}})}+{{\rm{FeO}}}_{({\rm{melt}})}+{{\rm{Al}}}_{2}{{\rm{O}}}_{3({\rm{melt}})}\\ \,=\,{({\rm{Di}}-{\rm{Hd}}-{\rm{CaTs}})}_{({\rm{cpx}})}+{{\rm{CO}}}_{2({\rm{vapour}})}\end{array}$$

5 $$\begin{array}{c}2{{\rm{CaCO}}}_{3({\rm{limestone}})}+{[2{{\rm{Al}}}_{2}{{\rm{O}}}_{3}+{{\rm{TiO}}}_{2}]}_{({\rm{magma}})}+{[{{\rm{CaFeSi}}}_{2}{{\rm{O}}}_{6}+{{\rm{MgFeSi}}}_{2}{{\rm{O}}}_{6}]}_{({\rm{cpx}})}\\ \,=\,{[{{\rm{CaAl}}}_{2}{{\rm{SiO}}}_{6}+{{\rm{CaFeAlSiO}}}_{6}+{{\rm{CaTiAlSiO}}}_{6}]}_{({\rm{cpx}})}\\ \,+{{\rm{Mg}}}_{2}{{\rm{SiO}}}_{4({\rm{ol}})}+2{{\rm{CO}}}_{2({\rm{vapour}})}\end{array}$$

6 $$\begin{array}{c}3{{\rm{CaCO}}}_{3({\rm{limestone}})}+{[{{\rm{SiO}}}_{2}+2{{\rm{Al}}}_{2}{{\rm{O}}}_{3}+{\rm{MgO}}+{{\rm{TiO}}}_{2}]}_{({\rm{magma}})}\\ \,+{[{{\rm{CaFeSi}}}_{2}{{\rm{O}}}_{6}+{{\rm{Mg}}}_{2}{{\rm{Si}}}_{2}{{\rm{O}}}_{6}]}_{({\rm{cpx}})}={[{{\rm{CaAl}}}_{2}{{\rm{SiO}}}_{6}+{{\rm{CaFeAlSiO}}}_{6}+{{\rm{CaTiAlSiO}}}_{6}]}_{({\rm{cpx}})}\\ \,+{[{{\rm{CaMgSiO}}}_{4}+{{\rm{Mg}}}_{2}{{\rm{SiO}}}_{4}]}_{({\rm{ol}})}+3{{\rm{CO}}}_{2({\rm{vapour}})}\end{array}$$

7 $$\begin{array}{c}4{{\rm{CaCO}}}_{3({\rm{limestone}})}+{{\rm{Al}}}_{2}{{\rm{O}}}_{3({\rm{melt}})}+4{{\rm{SiO}}}_{2({\rm{melt}})}={{\rm{CaAl}}}_{2}{{\rm{Si}}}_{2}{{\rm{O}}}_{8({\rm{pl}})}+{{\rm{Ca}}}_{2}{{\rm{Si}}}_{2}{{\rm{O}}}_{6({\rm{cpx}})}\\ \,+{{\rm{CaO}}}_{({\rm{melt}})}+4{{\rm{CO}}}_{2({\rm{vapour}})}.\end{array}$$

Equation (2) has been proposed by Iacono Marziano *et al*. (ref.^77^) modelling natural basalts from Stromboli; Eq. (3) is from Iacono Marziano *et al*. (ref.^75^) modelling the most primitive melt of Alban Hills (phono-tephrite); Eq. (4) is from Mollo *et al*. (ref.^78^) modelling a synthetic glass resembling the most primitive K-basalt of the Roman Province; Eqs (5 and 6) are from and Mollo and Vona (ref.^79^) modelling Mt. Vulsini shoshonite; Eq. (7) is from Carter and Dasgupta (ref.^80^) modelling a composition similar to that used by Iacono Marziano *et al*. (ref.^75^) and a Vesuvius shoshonite. Following these experimental evidences, some Alban Hills volcanic rocks have been interpreted as related to silicatic magma experiencing extensive carbonate assimilation (both limestone and dolostone) on the basis of the common presence of skarns and magmatic calcite^51,54,77^.

Other reactions include the increase of larnite component in olivine, but not the formation of pure monticellite^81^:

8 $$\begin{array}{c}4{{\rm{CaCO}}}_{3({\rm{limestone}})}+{[2{{\rm{Al}}}_{2}{{\rm{O}}}_{3}+{{\rm{TiO}}}_{2}]}_{({\rm{m}}{\rm{e}}{\rm{l}}t)}+{{\rm{Fe}}}_{2}{{\rm{SiO}}}_{4({\rm{ol}})}\\ \,+{[{{\rm{CaMgSi}}}_{2}{{\rm{O}}}_{6}-{{\rm{Mg}}}_{2}{{\rm{Si}}}_{2}{{\rm{O}}}_{6}]}_{({\rm{cpx}})}={[{{\rm{Mg}}}_{2}{{\rm{SiO}}}_{4}-{{\rm{Ca}}}_{2}{{\rm{SiO}}}_{4}]}_{({\rm{ol}})}\\ \,+{[{{\rm{CaAlAlSiO}}}_{6}-{{\rm{CaFeAlSiO}}}_{6}-{{\rm{CaTiAlSiO}}}_{6}]}_{({\rm{cpx}})}\\ \,+{[{\rm{MgO}}+{\rm{FeO}}]}_{({\rm{melt}})}+4{{\rm{CO}}}_{2({\rm{vapour}})}\end{array}$$

Also in this case, the major impact of carbonate (calcite/limestone) digestion on the mineralogy of hybrid magma is the increase of modal abundance of clinopyroxene and its increase in kushiroite (CaAl_2_SiO_6_), esseneite (CaFe^3+^AlSiO_6_) and grossmanite (CaTiAlSiO_6_) components in clinopyroxene. In the case of Polino, the absence of clinopyroxene and plagioclase, as well as the presence of a monticellite rim around liquidus Mg-Fe olivine and as groundmass phase indicate strongly SiO_2_ undersaturated compositions of the pristine (pre-carbonate contamination) mantle melt, suggesting its ultrabasic nature. The reaction we envisage can be simplified as follows:

9 $${{\rm{CaCO}}}_{3({\rm{limestone}})}+{{\rm{Mg}}}_{2}{{\rm{SiO}}}_{4({\rm{olivine}})}={{\rm{CaMgSiO}}}_{4({\rm{monticellite}})}+{{\rm{MgO}}}_{({\rm{melt}})}+{{\rm{CO}}}_{2({\rm{vapour}})}$$

Equation (9) was proposed by Walter (ref.^82^) and can explain the reaction observed in the rim of olivine phenocrysts in contact with monticellite. Another possible reaction could be represented by Bowen (ref.^83^):

10 $$2{{\rm{CaCO}}}_{3({\rm{limestone}})}+{{\rm{Mg}}}_{2}{{\rm{SiO}}}_{4({\rm{olivine}})}+{{\rm{CaMgSi}}}_{2}{{\rm{O}}}_{6({\rm{melt}})}=3{{\rm{CaMgSiO}}}_{4({\rm{monticellite}})}+2{{\rm{CO}}}_{2({\rm{vapour}})}$$

In their experiment (run 486) carried out in the CaO-MgO-SiO_2_-CO_2_-H_2_O system at 1038 °C and 2 kb, Otto and Wyllie (ref.^84^; p. 357) report “*clusters of subhedral crystals of monticellite with enclosed euhedral crystals of forsterite*” associated with subhedral mostly angular calcite crystals and ~20 wt% SiO_2_ in the experimental run. This paragenesis and the SiO_2_ content closely resemble that of Polino rocks, and is, therefore, compatible with a process of monticellite crystallization at shallow depths rather than invoking equilibrium with forsterite at P > 1 GPa, as instead proposed by literature models. In the Otto and Wyllie (ref.^84^) experiments, forsterite is always the major liquidus silicate mineral in low-SiO_2_ mixtures. With increasing SiO_2_ in the starting material, the liquidus minerals are olivine (melt SiO_2_ = 5–10 wt%), olivine and monticellite (melt SiO_2_ = 15–20 wt%), olivine and åkermanite or monticellite and åkermanite (melt SiO_2_ = 25–30 wt%), åkermanite and diopside (melt SiO_2_ = 30–35 wt%), diopside (melt SiO_2_ = 40 wt%) and diopside and quartz (melt SiO_2_ = 45–50 wt%; ref.^84^).

## Carbonatite Melt-Silicate Solid vs. Silicate Melt-Carbonatite Solid Constraints

The data presented above and those from previous experimental studies suggest a different origin for the CaO enrichment in Polino rocks, i.e. a shallow depth provenance rather than an upper mantle source. The strongly radiogenic ^87^Sr/^86^Sr (>0.710; ref.^85^) and unradiogenic ^143^Nd/^144^Nd (<0.5122) of Polino and other ULUD carbonatites and calcite separates overlap the ULUD silicatic rocks. These values are far from the field of worldwide carbonatites (^87^Sr/^86^Sr <0.7065; ^143^Nd/^144^Nd > 0.5123; refs^47,85,86^), questioning the interpretation of Polino calcite as a mantle carbonatitic component. The ^87^Sr/^86^Sr of Polino calcite and whole-rock carbonatite is also higher than that of local Calcare Massiccio limestone (<0.7075; ref.^85^), but this cannot be interpreted against a sedimentary origin of the Polino carbonate fraction. Indeed, in a hypothetical limestone-silicatic magma interaction, the Sr-rich ULUD magma (Sr up to 4000 ppm) strongly controls the variation of ^87^Sr/^86^Sr ratios in the contaminated melts. In this case, minimum amounts of magma can easily modify the original ^87^Sr/^86^Sr of the carbonate-rich fraction of the contaminated magma, being the sedimentary fraction characterized by more than one order of magnitude less Sr.

Dolostones are present in minor amount in the carbonate succession pierced by the magma and could be potentially responsible for the high Fo content of Polino olivines. According to Iacono Marziano *et al*. (ref.^75^), the magnesite end-member of dolomite reacts with SiO_2(melt)_ via:

11 $$2{{\rm{MgCO}}}_{3}+2{{\rm{SiO}}}_{2}={{\rm{Mg}}}_{2}{{\rm{SiO}}}_{4}+2{{\rm{CO}}}_{2}$$

Dolomite digestion often results in crystallization of MgO-rich olivine together with clinopyroxene, this latter being the only crystallizing phase in calcite-doped experiments^75^. The very low NiO of MgO-rich and CaO-rich composition of Alban Hills volcano olivines is considered not a mantle origin feature, but rather the effect of digestion of dolostone^43^, a conclusion confirmed by experimental data^75^. North Baikal olivines formed after digestion of dolostones by mafic magma are characterized by similar low NiO (<0.25 wt%; average 0.12 wt%)^87^. Polino olivines are characterized by high NiO (~0.06–0.95 wt%; average ~0.36 wt%, ~3300 ppm Ni), without any correlation between NiO and MgO (R^2^ = 0.06; Table A2). These and the previous considerations lead us to hypothesize for the Fo-rich composition of Polino olivines a mantle origin, rather than a process of interaction with dolostones. This conclusion is corroborated by the very homogeneous composition of the largest olivines (Table A2), indicating the absence of variation from the core to the near rim sectors. Only the outermost olivine rims show MgO increase, associated to the CaO increase, as consequence of interaction with limestone. This process leads Ca entering the M2 olivine site, leaving Mg free entering the M1 site, not preferred by Fe (ref.^81^).

As concerns the stable isotopes, the high δ^18^O (~+24‰; ref.^9^) of the calcite component cannot be used to infer a mantle origin for the carbonate component in Polino rocks. The low δ^13^C of Polino calcite (from −8.0 to −13.1‰; ref.^9^) is within the δ^13^C values of Alban Hills skarns (from +3 to −12‰; ref.^53^), Alban Hills lavas (from +5 to −19‰; ref.^53^) and the Morron de Villamayor sedimentary calcite entrapped in olivine (from −11 to −12‰; ref.^88^). Natural Alban Hills clinopyroxenes show good correlation between δ^18^O and ^IV^Al with increasing CaO content^51^. Experimental modelling of AFC process adding carbonate component to a silicatic magma shows the same compositional evolution of clinopyroxenes, with the highest kushiroitic component recorded in the experimental runs with higher amounts of added carbonates (e.g., refs^78,81^). If these stable isotope data cannot be used to definitively assume a sedimentary origin for the carbonate fraction of Polino rocks, they are at least compatible with such an origin.

As monticellite is known to be unstable at pressures >1 GPa (ref.^89^), the hypothesis of monticellite formation after the reaction of mantle minerals with Ca-carbonatitic melt below the Moho appears improbable, and implies its secondary origin. In other words, our view overturns the classically accepted interpretation^8,9^ of monticellite as the reaction product between a carbonatitic magma infiltrating mantle matrix, then scraped off by the same carbonatitic liquid. We believe, instead, that the interaction occurred between an ultrabasic melt and the sedimentary carbonate wall rocks *en route* to the surface.

The presence of phlogopite, forsterite, monticellite, perovskite and periclase (the first four phases identified in Polino rocks) is not a proof to discriminate a carbonate-rich rock as carbonatite, being these minerals present in impure marbles too^38,50,90^. Also, the presence of schorlomitic garnet in Polino rocks^59^ cannot be considered a proof for a carbonatitic origin of the melt, being this mineral a typical phase of impure limestones, marbles and skarns (e.g., refs^90–92^). According to several authors (refs^29,38,43,51^), no decisive chemical and mineralogical criterions by which to establish a magmatic heritage for a carbonate-rich rock do exist, considering that also magmatic carbonatite can undergo drastic compositional changes after emplacement.

Recognition of primary carbonate components must be based on the association with igneous rocks, the presence of fenites, euhedral crystals of carbonates evidencing flow structures, the mineral paragenesis, the incompatible element composition and the radiogenic and stable isotopic ratios of the carbonate-rich rocks^27^. None of these evidences can be found at Polino: (*1*) no silicate pair rocks are associated to the “carbonatitic” diatreme; (*2*) no fenitization is recorded on country rocks; (*3*) no euhedral tabular or rhombic crystals of calcite have been found; (*4*) no flow structures of carbonate materials have been identified; (*5*) monticellite stability field is constrained to shallow P (<1 GPa), rendering impossible the formation of this mineral at mantle depths; (*6*) monticellite appearance is contemporaneous with cessation of olivine growth and crystallization; (*7*) the incompatible and compatible element budget of Polino “carbonatite” is different from average worldwide Ca-carbonatites^62,63,93^ and Fort Portal monticellite carbonatite^68^; (*8*) Radiogenic isotopic ratios indicate interaction with crustal lithologies, while stable isotopic ratios are compatible with C-O exchanges at relatively low-T conditions.

## Conclusions

At Polino, plagioclase- and pyroxene-free rocks characterized by abundant Fo-rich olivine, monticellite, calcite, phlogopite, perovskite, Fe-Ti oxides were emplaced ~246 ka. Their whole-rock composition is intermediate between Ca-carbonatitic magma and alkali-poor ultrabasic melt.

On the basis of petrographic evidences (euhedral shape of olivine and phlogopite crystals, presence of monticellite as groundmass phase and as rim around olivine), mineral chemical compositions (homogeneous Fo-rich and modestly CaO-enriched olivine, high Mg# phlogopite), experimental constraints (stability field of monticellite limited to <1 GPa), isotopic data (strongly radiogenic ^87^Sr/^86^Sr, high δ^18^O, low δ^13^C) and geological-volcanological considerations (small volume of the magma, limited diameter of the diatreme, over-thickened sedimentary carbonate country-rocks pierced by the upwelling magma), we propose that Polino rocks cannot be considered as true carbonatites (monticellite alvikite), as instead reported in literature. The origin of these rocks is, rather, more compatible with interaction between an ultrabasic melt likely generated by a magnesite-bearing residual peridotite and local sedimentary carbonate rocks.

The classification of Polino volcanic rocks remains debated. It cannot be defined as alvikite (too low primary carbonate content), basalt (no plagioclase), kamafugite (no kalsilite), melilitite (no melilite), foidite (no foids), dunite (not a plutonic/metamorphic rock) or picrite (SiO_2_ <30 wt%). It represents an alkali-poor strongly ultrabasic melt whose original composition was modified by the digestion of sedimentary carbonates, and can consequentially be classified as pseudocarbonatite^30^. The final message is that the carbonate component of Polino rocks is not mantle-derived, but rather is the result of partial digestion of shallow limestones.

On the other hand, the original Polino magma (i.e., the composition before interaction with sedimentary carbonates) is considered to derive from a carbonated (magnesite-bearing) peridotitic source, to explain the ultrabasic compositions (absence of feldspars and clinopyroxene) and the Mg-rich olivine and phlogopite phenocrysts. In other words, the petrogenetic model proposed is a fuzzy one, based not on a carbonatite-yes or carbonatite-no choice. At the same time, the Polino rocks are not true carbonatites, but they derive from a carbonated mantle source.

## Supplementary information

Dataset Table A1 to A4
